# Postoperative atrial fibrillation does not impact on overall survival after esophagectomy in patients with thoracic esophageal cancer: results from a randomized, double-blind, placebo-controlled trial

**DOI:** 10.18632/oncotarget.27643

**Published:** 2020-06-23

**Authors:** Toshiyasu Ojima, Masaki Nakamura, Keiji Hayata, Junya Kitadani, Masahiro Katsuda, Mikihito Nakamori, Akihiro Takeuchi, Shimpei Maruoka, Naoki Fukuda, Shinta Tominaga, Hideki Motobayashi, Hiroki Yamaue

**Affiliations:** ^1^Second Department of Surgery, Wakayama Medical University, Wakayama, Japan

**Keywords:** esophageal cancer, atrial fibrillation, landiolol, randomized controlled trial, complication

## Abstract

Background: Administration of landiolol hydrochloride was found to be associated with reduced incidence of atrial fibrillation (AF) after esophagectomy for esophageal cancer in our previous randomized controlled trial (RCT). In addition, reduced incidence of AF was associated with reduction of other complications. Meanwhile, the effects of postoperative AF and other complications on long-term survival following esophagectomy are not well understood.

Materials and Methods: Between March 2014 and January 2016, 100 patients with esophageal cancer were registered in an RCT trial and randomly allocated to receive either administration of landiolol or a placebo. We analyzed data from this RCT to better understand the effect of postoperative AF and severe associated complications on overall survival (OS) after esophagectomy for cancer. We also examined whether prophylactic administration of landiolol hydrochloride directly affects prolonged survival in patients with esophageal cancer.

Results: The five-year rates of OS in the patients with and without AF were 60%, and 68.6%, respectively, there was no significant difference (*P* = 0.328). Five-year rates of OS of the patients with and without severe complications were 64.6%, and 67.5%, respectively (*P* = 0.995). The five-year rates of OS in the placebo and landiolol groups were 65.8% and 68%, respectively (*P* = 0.809). In multivariate analysis, high stage (stage III/IV) alone was an independent prognostic factor for esophageal cancer patients following esophagectomy.

Conclusions: New-onset AF and the other severe complications were not associated with poorer long-term survival following esophagectomy. In addition, administration of landiolol hydrochloride after esophagectomy did not contribute to prolonging the OS.

## INTRODUCTION

Esophagectomy is considered the optimum treatment against esophageal cancers. Despite improvements in surgical skills and the introduction of minimally invasive surgery, such as thoracoscopic esophagectomy, the overall rate of morbidity remains very high.

New-onset atrial fibrillation (AF) following esophagectomy occurs in 10–40% of patients with esophageal cancer [1–4]. Postoperative AF may directly increase the risk of thromboembolic events and is also known to indirectly increase rates of other complications, such as anastomotic leakage, pneumonia, and respiratory failure, through organ hypoperfusion [5–7]. The incidence of major postoperative complications in our previous study increased in patients that developed new-onset AF following subtotal esophagectomy [7]. Strategies targeting prevention of AF after esophagectomy may therefore be valuable.

Landiolol hydrochloride is an ultra-short acting beta 1-selective blocker that can be administered intravenously. It has a half-life of only four minutes and it has a weaker negative inotropic effect than other intravenous beta 1-blockers [8–10]. We performed a randomized, double-blind, placebo-controlled comparative phase III trial (UMIN000010648) to examine if intravenous administration of landiolol hydrochloride is effective in the prevention of AF after esophagectomy. We also examined if reduced incidence of AF may reduce other postoperative complications. Administration of landiolol hydrochloride had association in our previous study with reduced incidence of AF after esophagectomy for esophageal cancer. This was associated with reduction in other postoperative complications [8].

The influences of new-onset AF after esophagectomy on long-term survival have not been widely studied. Two papers reported no significant impact on three-year survival [4, 5]; while another two papers considered new-onset AF after esophagectomy to be an independent factor in poor prognosis [11, 12]. The effect of postoperative AF on long-term survival following esophagectomy is therefore controversial.

Severe postoperative complications may make patients with esophageal cancer less likely to survive over the long term. Patients with esophageal cancer but without severe postoperative complications have been shown to have better long-term survival than patients with complications [13–15]. Elsewhere, no such relationship was shown between severe complications and long-term survival [16–19].

The current study uses our randomized controlled trial (RCT) data to investigate the effect of postoperative AF on long-term oncological outcomes following transthoracic esophagectomy for cancer. We also evaluate the influence of severe postoperative complications on overall survival (OS) and whether prophylactic administration of landiolol hydrochloride directly influences prolonged survival in patients with esophageal cancer.

## RESULTS

### Patients characteristics and surgical outcomes

Table 1 shows patient characteristics. There were no significant differences between the two groups in age, gender, body mass index, percentage of patients receiving neoadjuvant chemotherapy, tumor location, pathological type of tumors, or Tumor-Node-Metastasis (TNM) stage. Table 2 shows surgical outcomes, with no differences between the two groups, which were well balanced.

**Table 1:** Clinicopathological characteristics of the patients

Variable	Placebo (*n* = 50)	Landiolol (*n* = 50)	*P* value
Clinical characteristics			
Age, years	69 (45–83)	68 (31–85)	0.782
Gender, Male/Female	41/9	36/14	0.235
BMI, kg/m^2^	22 (16–29)	22 (13–37)	0.837
Neoadjuvant chemotherapy	19 (38%)	27 (54%)	0.108
Docetaxel + Cisplatin + 5-FU	7	16	
Docetaxel + Cisplatin + S-1	12	11	
Pathological characteristics			
Tumor location, U/M/L	7/27/16	8/26/16	0.958
Pathological type (scc/adeno/other)	45/3/2	46/3/1	0.842
TNM stage^*^, I/II/III/IV	19/10/20/1	17/11/21/1	0.980

^*^Tumor-Node-Metastasis (TNM) Classification of the International Union Against Cancer (UICC), seventh edition. Abbreviations: BMI, body mass index; 5-FU, 5- fluorouracil; U, upper third thorax; M, middle third thorax; L, lower third thorax; scc, squamous cell carcinoma; adeno, adenocarcinoma.

**Table 2:** Surgical outcomes

Variable	Placebo (*n* = 50)	Landiolol (*n* = 50)	*P* value
Surgical outcomes			
Lymph node dissection, three-field/two-field^*^	16/34	19/31	0.529
Total duration of surgery, min	473 (311–606)	457 (355–609)	0.597
Blood loss, ml	70 (10–435)	115 (30–655)	0.065
Blood transfusion	4 (8%)	8 (16%)	0.357
R0 curative resection	48 (96%)	48 (96%)	0.999
Complications			
Atrial fibrillation	15 (30%)	5 (10%)	0.012
All complication, more than grade III^**^	16 (32%)	4 (8%)	0.003
Mortality	0	0	0.999

^*^Three-field indicates bilateral cervical regions, mediastinal spaces and abdomen; two-field indicates mediastinum and abdomen. ^**^Surgical complications were classified into five categories according to the Clavien-Dindo classification.

### Postoperative complications

New-onset AF was noted in 15 patients in the placebo group and in five patients in the landiolol group (Table 2). There were significant differences (*P* = 0.012). Overall incidence of postoperative complications of more than Clavien-Dindo Grade IIIa were significantly higher in the placebo group (32%) than in the landiolol group (8%) (*P* = 0.003).

### Recurrence of tumors

Median follow-up periods in the placebo and landiolol groups were 60.5 months and 58 months, respectively. The percentage of patients receiving adjuvant chemotherapy was similar between the two groups. Fourteen patients in the placebo group and 13 patients in the landiolol group had recurrence of esophageal cancer (*P* = 0.822, Table 3). Table 3 shows the details of the recurrent patients. These 27 patients received chemotherapy, chemoradiotherapy or palliative surgery, and four patients survive at the time of writing.

**Table 3:** Oncological outcomes

Variable	Placebo (*n* = 50)	Landiolol (*n* = 50)	*P* value
Follow-up periods, months	60.5 (4–83)	58 (6–84)	0.691
Adjuvant chemotherapy	10 (20%)	9 (18%)	0.799
S-1	2	3	
Paclitaxel	2	3	
Docetaxel + Cisplatin + S-1	2	1	
5-FU + Cisplatin	2	1	
5-FU + Cisplatin + radiation	2	0	
CPT-11 + Cisplatin	0	1	
Recurrence	14 (28%)	13 (26%)	0.822
First recurrence site,			
LN/lung/liver/bone/others	5/5/1/1/2^*^	7/3/2/1/0	
TNM stage^**^, I/II/III/IV	2/1/11/0	1/2/10/0	

^*^One skin metastasis and 1 brain metastasis. ^**^Tumor-Node-Metastasis (TNM) Classification of the International Union Against Cancer (UICC), seventh edition. Abbreviations: 5-FU, 5- fluorouracil; CPT-11, irinotecan; LN, lymph node.

### Follow-up data of the patients with stage I, II

Table 4 shows the recurrence rates and mortality rates in the patients with stage I and II. When limited to the patients with stage I and II, the recurrence rates in the patients with and without AF were 15%, and 9%, respectively (*P* = 0.611). However, overall mortality rate was significantly higher in the patients with AF (39%) than in the patients without AF (14%) (*P* = 0.046).

**Table 4:** Follow-up data of the patients with stage I, II

Variable	Patients with AF (*n* = 13)	Patients without AF (*n* = 44)	*P* value
Recurrence	2 (15%)	4 (9%)	0.611
Overall mortality	5 (39%)	6 (14%)	0.046
Cancer-specific mortality	2 (15%)	3 (7%)	
Mortality of other diseases	3 (23%)	3 (7%)	

Abbreviations: AF, atrial fibrillation.

### Survival outcomes

**Figure 1:**
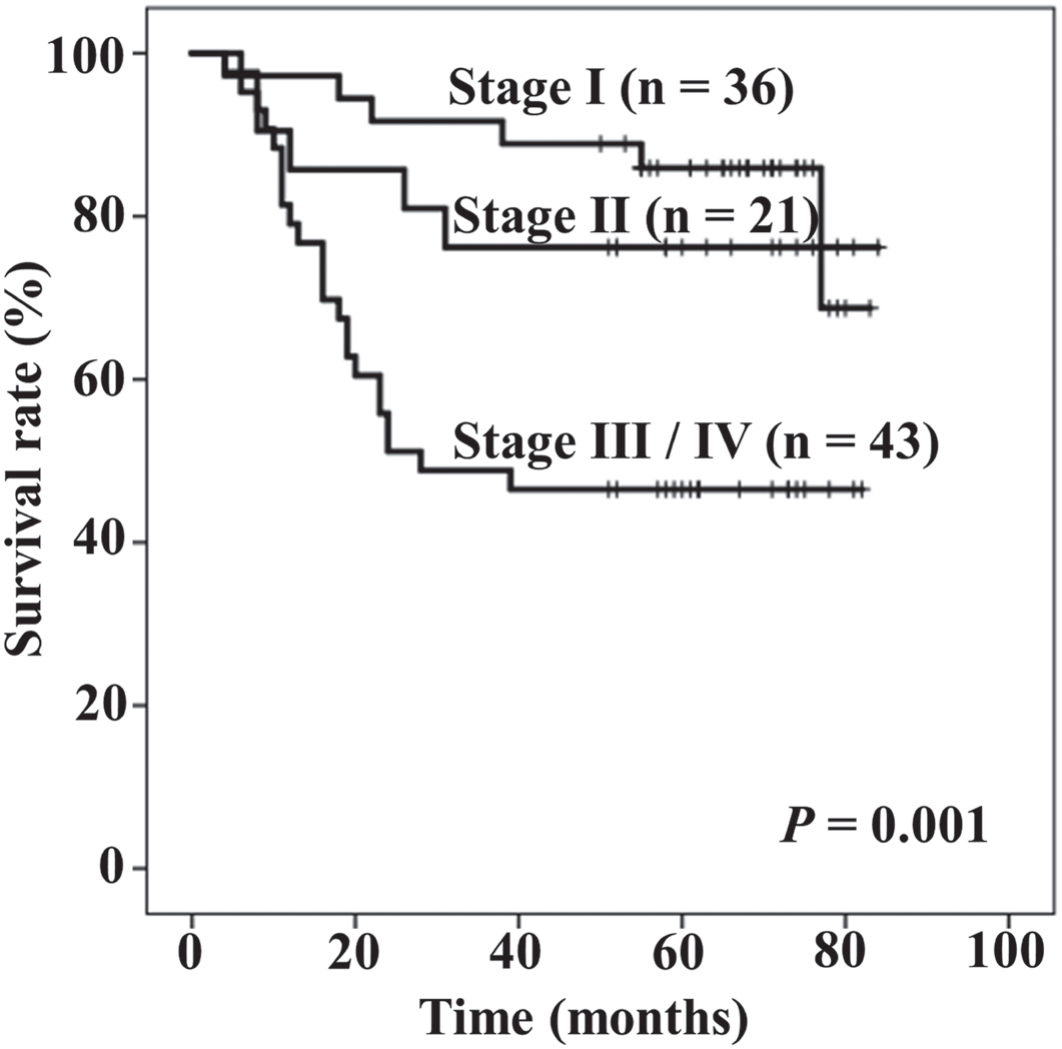
Kaplan–Meier curves of overall survival stratified by Tumor-Node-Metastasis (TNM) stage.

Figure 1 shows Kaplan–Meier curves of OS stratified by TNM stage. The five-year rates of OS in patients with stage I, II, and III / IV were 85.9%, 76.2%, and 46.5%, respectively (*P* = 0.001).

**Figure 2:**
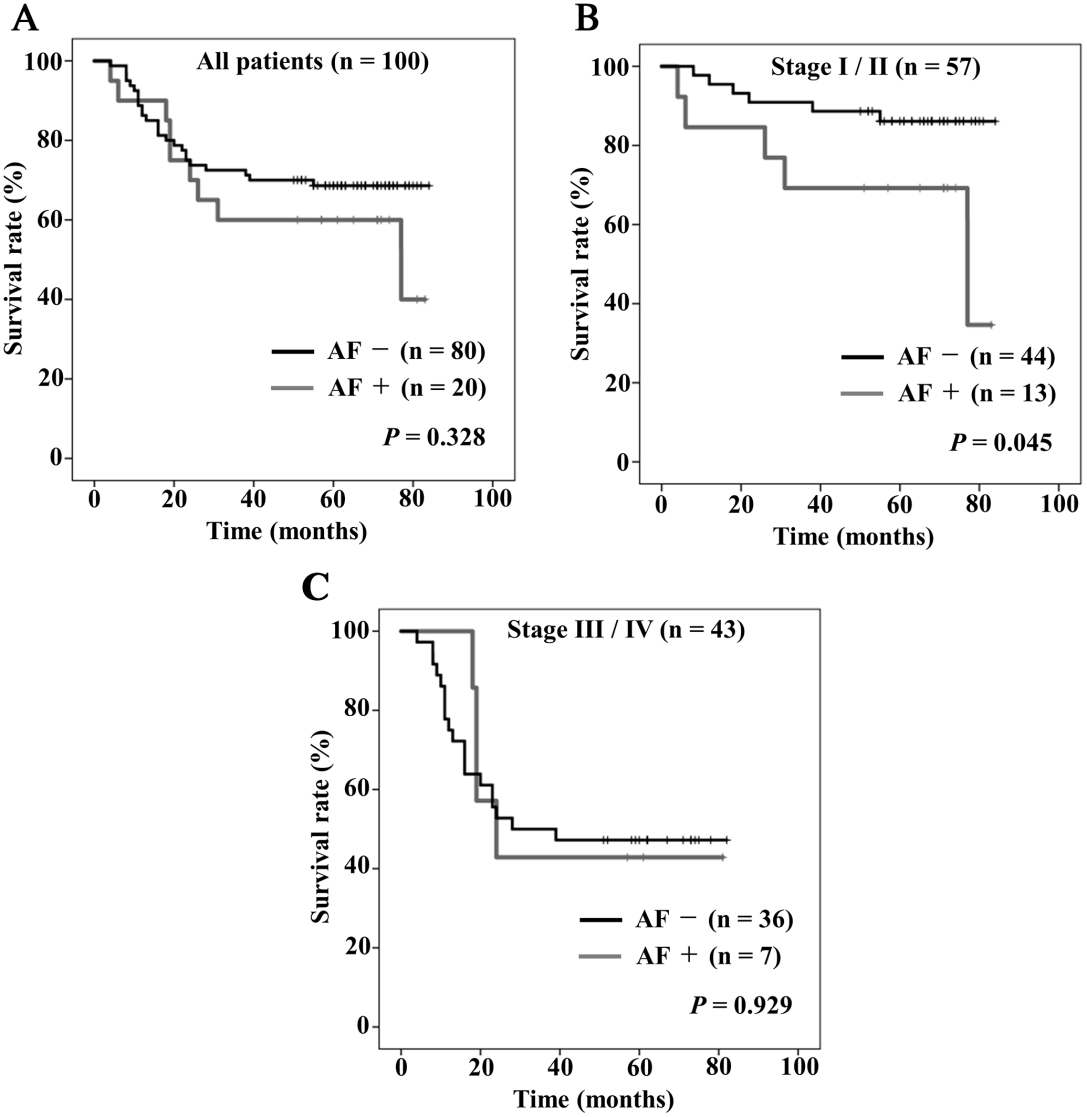
Kaplan–Meier curves of overall survival stratified by the presence (gray) or absence (black) of postoperative atrial fibrillation (AF). (**A**) All patients, (**B**) stage I/II, (**C**) stage III/IV.

Figure 2 shows Kaplan–Meier curves of OS stratified by the presence or absence of postoperative AF. The five-year OS rates in all patients with and without AF were 60%, and 68.6%, respectively, there were no significant differences in the OS rates between the two groups (Figure 2A, *P* = 0.328). In stage I/II stratification, there were significant differences in the rates of OS between the two groups (Figure 2B, *P* = 0.045), but in stage III / IV stratification there were no significant differences (Figure 2C, *P* = 0.929).

**Figure 3:**
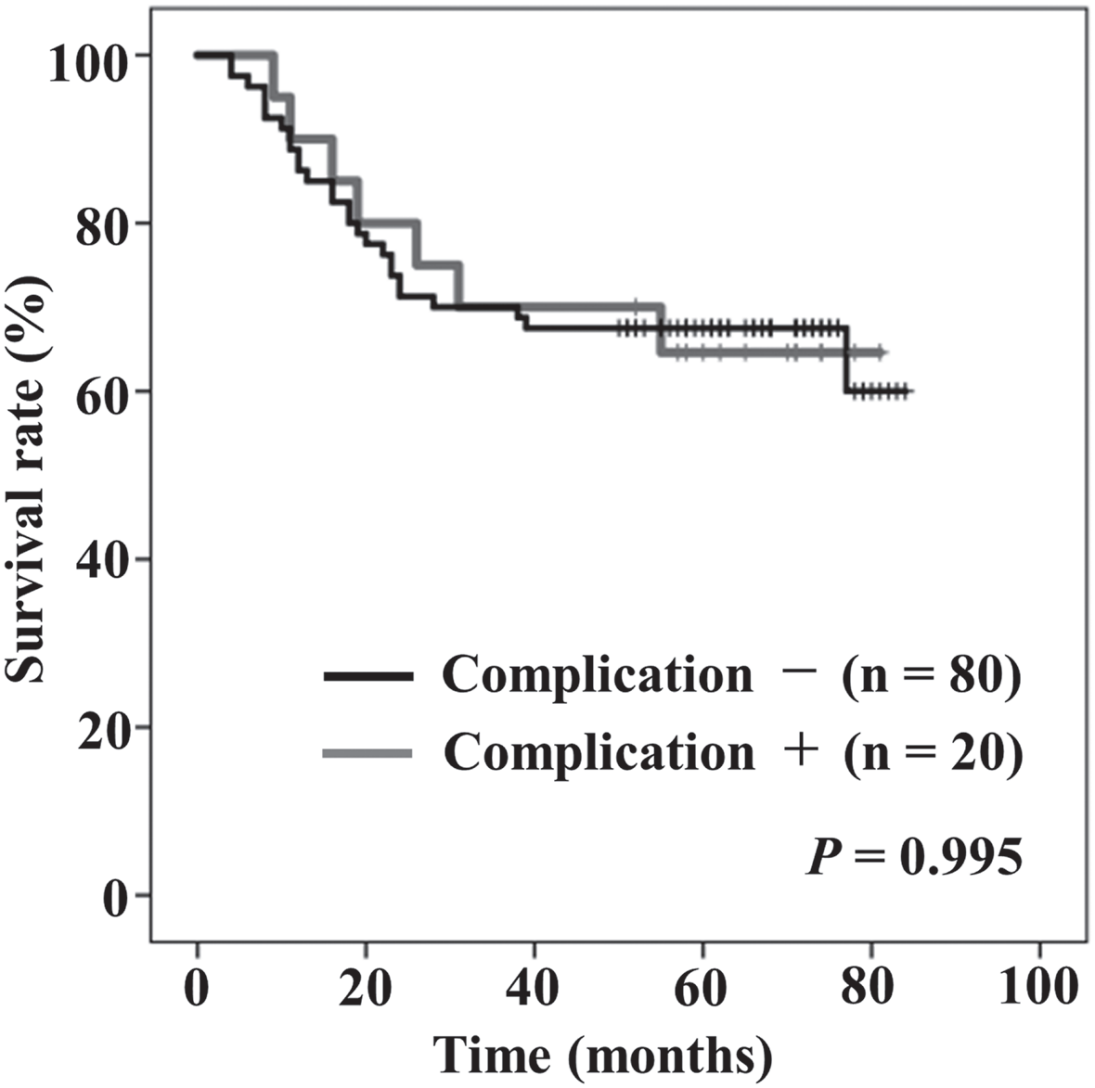
Kaplan–Meier curves of overall survival stratified by the presence (gray) or absence (black) of postoperative complication (more than Clavien-Dindo classification grade IIIa).

Figure 3 shows Kaplan–Meier curves of OS stratified by the presence/absence of postoperative complications (more than Clavien-Dindo grade IIIa). Five-year rates of OS in the patients with and without postoperative complications were 64.6%, and 67.5%, respectively. There was no significant difference in the rates of OS between the two groups (*P* = 0.995).

**Figure 4:**
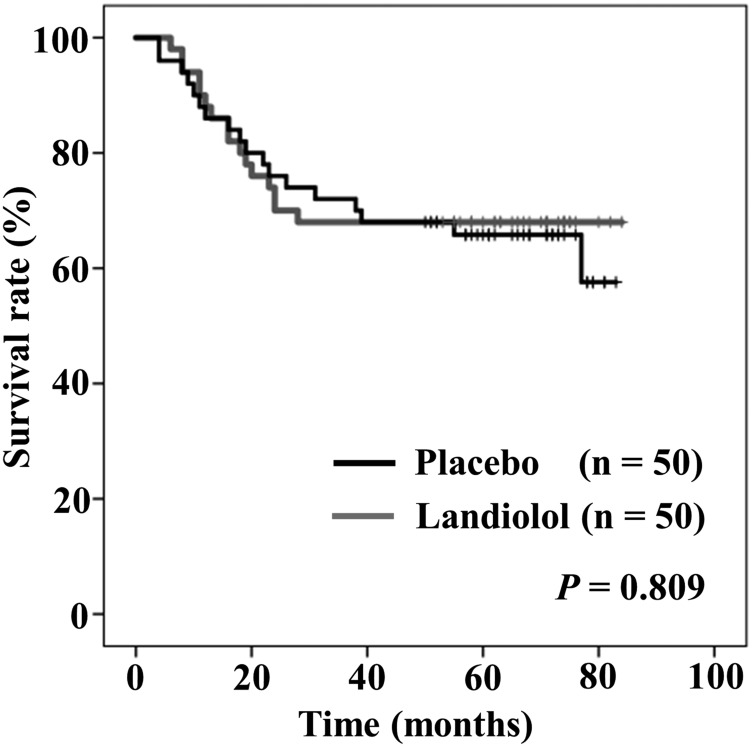
Intention to treat Kaplan–Meier curves of overall survival between placebo (black) and landiolol (gray) groups.

Figure 4 shows intention to treat Kaplan–Meier curves of OS between the placebo and the landiolol groups. Five-year OS rates in the placebo and landiolol groups were 65.8%, and 68%, respectively. There was no significant difference in the rates of OS between the two groups (*P* = 0.809).

### Univariate and multivariate Cox proportional hazard model analysis for OS

Univariate and multivariate OS analyses were calculated using Cox proportional hazard regression model. In univariate analysis, 3 of the 13 factors decreased OS in patients with esophageal cancer following esophagectomy (Table 5, *P* < 0.10). The factors were diabetes, patients who underwent curative resection, and patients with TNM stage III/IV. Multivariate analysis revealed that among patients with esophageal cancers following esophagectomy, only the patients with TNM stage III/IV (*P* = 0.001; hazards ratio, 3.413) were independent associative factors for decreased OS (Table 5).

**Table 5:** Univariate and multivariate Cox proportional hazard model analysis for overall survival

Risk factors	Categories	Univariate analysis		Multivariate analysis	
		*P*	Hazards ratio (95% CI)	*P*	Hazards ratio (95% CI)
Hypertension	yes	0.479	0.781 (0.394–1.548)		
	no				
Diabetes mellitus	yes	0.022	2.369 (1.131–4.961)	0.067	2.067 (0.950–4.496)
	no				
Old myocardial infarction	yes	0.981	0.976 (0.133–7.164)		
	no				
COPD	yes	0.153	0.421 (0.129–1.379)		
	no				
Smoking	yes	0.346	1.533 (0.631–3.727)		
	no				
Daily drinker	yes	0.990	0.994 (0.411–2.403)		
	no				
Ejection fraction	60% <	0.870	1.066 (0.497–2.286)		
	60% ≥				
TNM stage^*^	III/IV	0.001	3.647 (1.771–7.509)	0.001	3.413 (1.633–7.135)
	I/II				
Blood transfusion	yes	0.526	1.360 (0.526–3.517)		
	no				
R0^*^ curative resection	yes	0.052	3.258 (0.989–10.731)	0.596	1.409 (0.397–5.002)
	no				
Postoperative atrial fibrillation	yes	0.334	1.457 (0.679–3.124)		
	no				
Postoperative complications (more than Grade IIIa^**^)	yes	0.995	0.997 (0.434–2.291)		
	no				
Assignment group	Placebo	0.810	0.921 (0.469–1.809)		
	Landiolol				

^*^Tumor-Node-Metastasis (TNM) Classification of the International Union Against Cancer (UICC), seventh edition. ^**^Surgical complications were classified into five categories according to the Clavien-Dindo classification. Abbreviations: CI, confidence interval; COPD, chronic obstructive pulmonary disease.

## DISCUSSION

In our prospective RCT, administration of landiolol was associated with reduction in incidence of new-onset AF following esophagectomy for esophageal cancer. Furthermore, reduction in the incidence of AF related to landiolol administration was associated with reduction in the incidence of the other severe postoperative complications [8].

New-onset AF was unrelated, however, to poorer long-term survival after esophagectomy. When limited to stage I and II patients with esophageal cancer, the occurrence of AF after esophagectomy may have an association with poor survival. Therefore, the prophylactic administration of landiolol hydrochloride may prolong the overall survival in patients with stage I and II. Contrary to previous reports [13–15], overall severe complications were not associated with long-term survival of these patients. This study refuted the hypothesis that administration of landiolol hydrochloride leads to improvement of long-term oncological outcomes after esophagectomy for esophageal cancer. Only high stage of cancer (stage III/IV) was an independent prognostic factor for patients with esophageal cancer following esophagectomy.

New-onset AF after esophagectomy is due to the preexisting cardiac substrate conducive to the activation of AF. This reflects long-standing cardiac remodeling and represents overall cardiovascular risk independent of acute onset of postoperative AF [12, 20]. Postoperative AF may therefore be correlated with long-term survival following esophagectomy [11, 12]. This hypothesis was not substantiated in the current study, however, which included patients in all stages. Patients with AF after esophagectomy may have increased risk of chronic AF in the future, and may have increased post-operative mortality, such as by thromboembolism, stroke or heart failure [12, 20]. In the current study, however, none of the patients who had new-onset AF after esophagectomy developed chronic AF. To better understand the relationship between new-onset AF after esophagectomy and long-term survival, clarification by large-scale multicenter clinical trials is required.

The influence of severe postoperative complications on the long-term survival of patients with esophageal cancer remains controversial [13–19]. In our results, there were no differences in survival rates between patients with and without severe complications. Severe complications, such as anastomotic leakage, pulmonary complications and bilateral vocal code paralysis, may impact on short-term surgical outcomes through a pathway of systemic inflammatory response. Long-term survival, however, may not be affected after the short-term surgical effect has disappeared [16]. There were no mortalities during this RCT, which was based in a high-volume center. In addition, the rate of severe complication (more than Clavien-Dindo grade IIIa) was low at 20%. All patients went through the same standardized multidisciplinary team process and were treated in a high-volume center. In general, patients with esophageal cancer who undergo esophagectomy in high-volume centers have been shown to have improved short- and long-term outcomes [15].

Invasion due to esophagectomy induces an increase in inflammatory cytokines. Perioperative inflammation is generally accepted to be related to tumor recurrence [8, 21, 22]. Landiolol hydrochloride has strong anti-inflammatory effects, it therefore not only reduces postoperative complication rates, but may also help to reduce tumor recurrence after esophagectomy [8]. Indeed, beta-blockers in general have been reported to improve prognosis for various cancers [23–27]. The anti-tumor effect exerted by beta-blockers may be mediated by suppression of the sympathetic nervous system through blocking of catecholamines [22]. This RCT has already proven a significantly lower serum IL-6 level in the landiolol group than in the placebo group during the early postoperative period [8]. In addition, landiolol hydrochloride may inhibit attachment of circulating tumor cells to vascular endothelial cells [21, 28]. In this study, however, the recurrence and survival rates were similar with and without administration of landiolol. In addition, there was no significant difference in the rates of relapse-free survival between the two groups (log-rank *P* = 0.661, data not shown). In our study, 3 μg/kg/min dose of landiolol was administered only during the 72 hr after esophagectomy. In previous papers, beta blockers improved prognosis for various cancers, but were administered over a long-term period [23–27]. To confirm whether prophylactic administration of landiolol directly influences prolonged survival in patients with esophageal cancer after esophagectomy, the dose and the duration of landiolol require examination.

This study had several limitations. First, due to the relatively small sample size (*n* = 100), findings from this study could not establish definitive evidence. It was also a single-center study. Multi-center large scale prospective RCT to evaluate whether intravenous administration of landiolol hydrochloride affects long-term survival after esophagectomy for esophageal cancer is therefore required. A second limitation is that the median follow-up period in this study was 60 months, so a longer follow-up period is needed. Thirdly, subsequent treatment after esophageal cancer recurrence was not defined, so differences in treatment after recurrence may have affected survival analysis.

In conclusion, new-onset AF and other severe complications were not associated with poorer long-term survival after esophagectomy. In addition, administration of landiolol hydrochloride after esophagectomy did not contribute to prolonged OS of patients with esophageal cancer.

## MATERIALS AND METHODS

### Study population

Participants of this study were patients with diagnosed esophageal cancer at the Wakayama Medical University Hospital (WMUH). Detailed inclusion criteria are described in our previous paper [8]. This was a randomized, double-blind, placebo-controlled comparative phase III trial [8], conducted in accordance with a protocol reviewed and approved by the WMUH Ethical Committee on Human Research (approval number 1235). The study protocol was registered at the University Hospital Medical Information Network (UMIN000010648).

### Randomization and blinding

Between March 2014 and January 2016, the 100 patients registered in the study were randomly allocated to receive administration of either landiolol or a placebo. After transthoracic esophagectomy with systematic lymphadenectomy, patients were randomized on postoperative day 1 in accordance with the study protocol. Infusion of landiolol hydrochloride (Ono Pharmaceutical, Osaka, Japan) was started at 3 μg/kg/min for 72 hours. Glucose solution was administered at 3 mL/hr during the same period. Patients, medical staff and investigators were all blinded to the treatments except one clinical research coordinator in the central registry and two selected pharmacists in WMUH who were aware of the treatments.

### Study endpoints

The primary end point of this RCT was the incidence of AF after esophagectomy. Secondary endpoints were incidence of all postoperative complications and OS.

AF was defined as an absent P wave before the QRS complex, with irregular ventricular rhythm shown on 12-lead electrocardiogram [3, 7, 8]. Occurrence of postoperative AF was defined as persistence of this arrhythmia for five minutes or more. Diagnosis of AF was re-confirmed by cardiologists. Postoperative complications were analyzed according to Clavien-Dindo classification [29].

### Follow-up and additional treatments

For stage I/II patients who underwent R0 resection, follow-up with semiannual endoscopy and abdominal computed tomography were performed. We used adjuvant chemotherapy for patients that were higher than stage III. For patients who had undergone R1 resection, additional chemoradiotherapy was recommended, but we chose the same kind of follow-up for cases where this was considered to be impossible because of severe coexisting diseases. Subsequent treatment was not defined in the case of recurring esophageal cancer.

### Statistical analysis

SPSS 22.0 software program (SPSS Inc., Chicago, IL) was used for all statistical analyses. Quantitative results are expressed as medians and ranges. Statistical comparisons between the placebo group and the landiolol group were performed with χ^2^ statistics and Fisher’s exact test; *P* < 0.05 was considered significant. OS was defined as the time from the esophagectomy to the date of death from any cause. Survival curves were generated using the Kaplan-Meier method and compared using the log-rank test; *P* < 0.05 was considered significant. Univariate and multivariate Cox proportional hazard models were used to evaluate factors that independently affected OS. Factors associated with univariate *P* < 0.10 were included in the multivariate analysis, while factors associated with multivariate *P* < 0.05 were defined as independent factors associated with OS.

## Abbreviations

AF: atrial fibrillation
RCT: randomized controlled trial
OS: overall survival
TNM: Tumor-Node-Metastasis
WMUH: Wakayama Medical University Hospital
